# On the Relative and Absolute Quantity of Carbonic Acid Exhaled by Man in Health and Disease

**Published:** 1846-07

**Authors:** 


### Art. XVI.

*De Quantitate relativâ et absolutâ Acidi Carbonici, ab Homine sano et ægroto exhalati.* Auctore Adolpho Hannover, Med. Lic., Med. secund. nosocomii Regii Fredericiani, &c. &c.—*Hafniæ,* 1845.

*On the relative and absolute Quantity of Carbonic Acid exhaled by Man in Health and Disease.* By Adolphus Hannover, m.d., &c.—*Copenhagen,* 1845. 8vo, pp. 91.

The object which Dr. Hannover has proposed to himself, in the investigations detailed in this little work, has been to compare the amount of carbonic acid exhaled by patients suffering under certain diseases,—to wit, phthisis, chronic bronchitis, and chlorosis,—with the healthy standard. Towards the determination of the latter he does not afford any new data; but contents himself with bringing together, and comparing, the results obtained by Allen and Pepys, Lavoisier and Seguin, Dr. Prout, and others among the earlier experimenters, with those of Andral and Gavarret, Professors Scharling, Valentin and Brenner, and Vierordt, in more recent times. The following summary affords a useful digest of the conclusions to which these combined results seem fairly to lead, with the authorities for each statement.

"1. The relative and absolute quantity of carbonic acid exhaled varies in different individuals, and in the same individual under different conditions.

"2. In both sexes the absolute quantity is governed according to the age. (Scharling, Andral, and Gavarret.)

"3. A greater absolute amount is exhaled by the male than by the female (Scharling) at every period of life, from the age of eight years to old age; but the difference is peculiarly obvious between the ages of sixteen and forty, when the male usually exhales nearly double the quantity exhaled by the female. (Andral and Gavarret.)

"4. In the male the absolute quantity increases from the age of eight years to that of thirty, and undergoes a sudden increase at the time of puberty; from the age of thirty the quantity diminishes, and this in proportion to the advance of age. (Andral and Gavarret.)

"5. In the female, also, the quantity increases up to the time of puberty, but, as soon as menstruation is established the quantity remains unchanged; whilst, on its cessation, the quantity suddenly undergoes a distinct increase, and then diminishes with the advance of age. During pregnancy the absolute amount rises to the standard of the non-menstruating female. (Andral and Gavarret.)

"6. In either sex the absolute quantity has a simple relation to the vigour of the bodily constitution, and to the evolution of the muscular system. (Andral and Gavarret.)

"7. In proportion to the weight of the body the infant exhales the largest absolute quantity, and the youth more than the middle-aged man. After the age of forty the quantity decreases, and the female seems to exhale a considerable amount in proportion to the weight of her body. (Scharling, Andral, and Gavarret.)

"8. The relative and absolute quantity increases shortly after food has been taken, and whilst digestion is going on; it is greater in the state of satiety than in that of fasting. (Prout, Valentin and Brunner, Lavoisier and Seguin, Scharling, Vierordt.) Imperfect digestion diminishes the absolute quantity. (Lavoisier and Seguin, Scharling.) After the use of alcohol in any form and quantity, especially when the stomach is empty, and even after the use of strong tea, the relative and absolute quantity becomes less. (Prout, Vierordt.)

"9. A moderate amount of muscular movement increases the relative and absolute amount of carbonic acid exhaled. (Prout, Scharling, Vierordt.)

"10. Depressing passions of the mind diminish the relative quantity. (Prout.)

"11. Frequent and deep respiratory movements augment the absolute quantity of carbonic acid exhaled (Allen and Pepys, Andral and Gavarret, Vierordt), but they diminish the relative amount. (Vierordt.) The frequency of the pulse seems to be of no consequence.

"12. The relative quantity is increased by the repeated inspiration of the same air, whilst the absolute is diminished. (Allen and Pepys, Scharling, Vierordt.) The absolute quantity appears to be increased by the respiration of pure oxygen. (Allen and Pepys, Nysten.) When the respiratory movements are impeded, or are altogether brought to a close, the relative quantity of carbonic acid contained in the air of the lungs is increased. (Vierordt.)

"13. By night and during sleep the absolute and relative qnantity is less than during the day and in the waking state (Prout, Scharling); the relative quantity is less in the morning than in the evening (Brande); but, before noon, the relative and absolute quantity is greater than in the evening. (Prout, Vierordt.)

"14. The absolute quantity is greater in winter (Lavoisier and Seguin); the relative is increased by electricity and low barometric pressure (Prout, Vierordt), but the absolute is diminished by greater rarity of the air. (Vierordt.)

"15. An increase of temperature diminishes both the relative and absolute quantity. (Vierordt.)

"16. The transpiration of carbonic acid through the skin is small in amount; the greatest activity in this function being in a vigorous adult male (Scharling)." (pp. 33-5.)

The whole subject of the cutaneous transpiration of carbonic acid seems to us to require much more careful consideration than it has yet received. We are inclined to believe that the cutaneous respiration is a very important adjunct to the pulmonary; and, in particular, that it may become greatly increased in amount in certain diseased states, when the pulmonary respiration is imperfectly performed. The only way to ascertain the truth on this subject, would be to employ such a closed chamber as that constructed by Professor Scharling, into which the product of the cutaneous respiration might freely pass; whilst the pulmonary respiration during the same period should be measured by a distinct apparatus, such as that employed by Andral and Gavarret, or by Valentin and Brunner; and to subject to this test not merely healthy subjects, but others suffering under various morbid conditions, especially such as produce obstruction to the due action of the lungs.

A larger number of experiments, moreover, seems yet wanting to determine the limits of variation in the absolute quantity of carbonic acid exhaled under different circumstances, as regards temperature, exercise, &c. This is a subject to which the attention of our arctic and antarctic voyagers might be advantageously directed, as well as that of residents in climates where the external temperature occasionally equals that of the body itself. As an average statement of the total quantity of carbon exhaled by a male adult of ordinary vigour, and leading a life of moderate activity, we think that the estimate of Scharling is entitled to the greatest weight; the amount deduced from his experiments on respiration in a closed chamber being 4080 grains, or 8½ oz. per day; whilst the estimate formed upon a calculation of the carbon contained in the food and in the excretions of a body of sailors (in whom we should expect that a larger exhalation would take place, conformably with the muscular exertions they employ, and the vicissitudes of temperature to which they are exposed), makes the daily excretion of carbon in a gaseous form no more than 5040 grains, or 10½ oz. And as this last estimate is founded, not upon the actual amount of provisions consumed, but upon the amount given out, of which a considerable part is wasted, it seems next to certain that some error must exist in the calculations of Liebig, who gives 6672 grains, or nearly 14 oz., as the average quantity of carbon daily exhaled by each individual of a body of soldiers in garrison.

Our information as to the amount of carbonic acid, whether relative or absolute, exhaled in morbid conditions of the body, is very imperfect. The researches of Nysten seem to lead to the following conclusions:

"1. In chronic diseases, without fever or pulmonary disorder, the chemical phenomena of respiration differ but little from those of normal condition.

"2. The air expired in severe continued fevers sometimes appears to contain a slightly-increased proportion of carbonic acid; but new experiments are required to determine whether this is a constant or an accidental occurrence.

"3. In certain diseases the expired air contains an unusually small proportion of carbonic acid; this is especially the case in severe dyspnœa, whether this result from degeneration of the tissue of the lungs, or from hydrothorax, ascites, or other causes interfering with their due expansion." (pp. 35-6.)

The well-known observations of Macgregor, relative to the decided increase in the amount of carbonic acid exhaled from the lungs during the acute stage of smallpox, measles, and scarlatina, and upon the augmentation which was presented in a fatal case of extensive icthyosis, are well known; but we think it doubtful whether this increase was due to an absolute increase in the quantity of carbonic acid exhaled, or whether it was not rather referrible to the stoppage of the cutaneous respiration, which would throw more work upon the lungs.

We have now to notice the researches of Dr. Hannover, which were carried on in conjunction with Professor Scharling. We hope that these are to be taken but as an earnest of more extended observations; since at present the amount of information contributed by them is very limited. In four cases of chlorosis, the amount of carbonic acid exhaled appeared to be above the average of that of menstruating females of corresponding age and bodily development; thus harmonizing with the statement of Andral and Gavarret; but this remarkable fact presented itself in addition, that an increase in the number of respirations per minute diminished, not merely the amount of carbonic acid exhaled at each expiration, but the total amount given off per minute. Thus the number of respirations per minute being, respectively— 17·3, 20·4, 25, and 37·3, the hourly exhalation of carbonic acid was—123·6, 118·6, 116·9, and 106·3 grs. thus showing a regularity of diminution corresponding with the increase of frequency, which could be scarcely attributed to other circumstances, more especially as the remarkably increased frequency in the last case was accompanied by a strongly-marked diminution of the exhalation.

In five cases of phthisis, a decided diminution was apparent in the total amount, excepting when exercise taken a short time previously had greatly augmented the rate of the respiratory movements,—this augmentation not being accompanied by a diminution, as in the preceding case, but by an increase in the amount of carbonic acid exhaled. The diminution, as compared with the healthy standard, was absolute, not only in itself, but also when due allowance was made for the loss of weight in the diseased state.

In two cases of chronic bronchitis, there was no decided departure from the normal standard. The same may be stated as to two cases of jaundice, one case of morbus cordis, and one case of morbus Brightii. A case of lithiasis is added, in which the respiration seemed much below the normal standard; by a strange omission, however, although a short summary of the case is given, no information is afforded as to the chemical nature of the calculi which were voided whilst the patient was under observation.

Besides the details of his experiments, Dr. Hannover propounds some interesting speculations founded upon their results, with regard to the connexion of the respiratory function with different states of the system, natural and morbid. Into these, however, we shall not follow him; since it appears to us that a much larger mass of data must be collected, before it is safe to begin to build upon them. We may remark, however, that these results, so far as they go, afford additional evidence against the assertion of Liebig, that phthisis is a disease of increased oxidation,—the very reverse being apparently the case. We need, however, to be informed, whether there is any increase in the cutaneous respiration in the advanced stages of this disease, when a large portion of the lungs is rendered unfit for the discharge of their function, and when the temperature of the body is sustained at its normal standard, or even higher. Again, the marked augmentation in the exhalation of carbonic acid in chlorosis, taken in connexion with the diminished amount of red corpuscles in the blood, in that disease, seems adverse to the view of Liebig, that the red corpuscles are the chief carriers of oxygen in arterial blood, and of carbonic acid in venous. We apprehend, however, that this increased exhalation of carbonic acid must be regarded as compensating for the deficiency of other secretions, and not as indicating any unusual activity in those interstitial changes to which the act of respiration is subservient.

